# Cathelicidin Mediates an Anti-Inflammatory Role of Active Vitamin D (Calcitriol) During *M. paratuberculosis* Infection

**DOI:** 10.3389/fcimb.2022.875772

**Published:** 2022-04-04

**Authors:** Joseph A. Vaccaro, Ahmad Qasem, Saleh A. Naser

**Affiliations:** Division of Molecular Microbiology, Burnett School of Biomedical Sciences, College of Medicine, University of Central Florida, Orlando, FL, United States

**Keywords:** vitamin D, cathelicidin, calcitriol, LL-37, MAP, CAMP, paratubercolosis, Crohn’s disease

## Abstract

Vitamin D is a key regulator in calcium and phosphorus metabolism which are essential for maintaining bone health. Recent reports also showed a role for vitamin D in immune regulation which may be linked to vitamin D deficiency in autoimmune disorders including inflammatory diseases and Crohn’s disease (CD). This study examines the role of vitamin D deficiency in the regulation of Cathelicidin Antimicrobial Peptide (*CAMP*) in CD-like macrophages. The latter includes macrophages infected with *Mycobacterium avium* subsp. *paratuberculosis* (MAP) isolated from CD patient. Initially, we measured cathelicidin and calcitriol in *ex vivo* plasma samples from CD patients with or without MAP infection (*N=40* per group). We also measured the expression and production of *CAMP*/LL-37, TNF-α, IL-1β, IL-10, cellular oxidative stress markers, and bacterial viability following treatment of MAP-infected macrophages with four different forms of vitamin D (D2, D3, calcifediol, and calcitriol). From these studies, we determined that LL-37 and calcitriol were significantly lower in CD samples from MAP-positive patients [155.55 ± 49.77 ng/mL and 51.48 ± 31.04 pg/mL, respectively] compared to MAP-negative patients [193.01 ± 78.95 ng/mL and 272.36 ± 94.77 pg/mL, respectively]. Moreover, calcitriol and calcifediol upregulated *CAMP* expression by nearly 5-fold and 3-fold, respectively. However, following MAP infection, only calcitriol increased *CAMP* by 3-folds. Both calcitriol and LL-37 reduced intracellular MAP viability by ~3 folds and inhibited TNF-α and IL-1β expression and production in these cells. Treating co-culture of Caco-2 monolayers and MAP-infected macrophages with LL-37 or calcitriol have shown a reduction in *NOX-1* expression and DHE signal, in addition to a higher NADPH/NADPt ratio. Notably, calcitriol’s anti-inflammatory effects were lost upon *CAMP* knockdown by CAMP-siRNA transfection. Altogether, the data indicate that MAP infection and burden is significant in CD by disrupting the conversion of calcifediol to calcitriol and downregulation of *CAMP* expression leading to vitamin D deficiency.

## Introduction

Vitamin D is a steroid hormone crucial to the efficient uptake and storage of calcium and phosphorus (Christakos et al., 2016). Most vitamin D are endogenously synthesized in humans using exposure to ultraviolet radiation, which converts 7-dehydrocholesterol to an isomer of the pro-vitamin D_3_; it then undergoes hydroxylation in the liver *via* the enzyme CYP27A1 to yield calcifediol or 25(OH)D_3_ (El-Sharkawy and Malki, 2020). Calcifediol makes up the majority of circulating vitamin D but displays minimal hormonal activity (Chang and Lee, 2019). When blood calcium or phosphate levels are low, the parathyroid gland detects the decline and releases parathyroid hormone (PTH) (Chang and Lee, 2019). PTH acts upon the kidneys to stimulate the hydroxylation of 25(OH)D_3_ to 1,25(OH)_2_D_3_, or calcitriol, using the enzyme CYP27B1 (Chang and Lee, 2019; El-Sharkawy and Malki, 2020). Interestingly, this enzyme can also be found in extra-renal tissues, including macrophages, where it regulates various intracellular events (Adams et al., 2014). Calcitriol, the active form of vitamin D, is then carried through the circulation to tissues across the body (Christakos et al., 2016). As a fat-soluble hormone, it is capable of binding to the ubiquitously expressed vitamin D receptor (VDR), which heterodimerizes with the retinoid X receptor (RXR), then translocates to the nucleus, where it begins stimulating transcription of its target genes (Christakos et al., 2016).

Among the genes enhanced by the VDR/RXR complex is *CAMP*, encoding Cathelicidin Antimicrobial Peptide (Carlberg, 2019). The active form of cathelicidin is LL-37, which is a 37 residue-long peptide produced by macrophages in response to inflammation (Vandamme et al., 2012). Like the defensin family, cathelicidin displays potent bactericidal and anti-inflammatory effects, through disruption of microbial membranes and conveying anti-inflammatory signals to immune cells (Vandamme et al., 2012). Cathelicidin has shown notable beneficial effects even on persistent, long-term infections like tuberculosis and those found in inflammatory bowel disease (IBD) (Liu et al., 2006; Liu et al., 2007; Tabatabaeizadeh et al., 2018; Alqasrawi et al., 2020; Alqasrawi et al., 2021). Its broad-spectrum effect on immunity makes cathelicidin a potential link between vitamin D and resistance to pathogens, even pathogens that are comparatively understudied; however, its dependence on vitamin D signaling exposes cathelicidin to disruption when calcitriol is restricted (Liu et al., 2007; Carlberg, 2019). Under ordinary circumstances, Toll-like receptors (TLRs) stimulation enhances transcription of the VDR and CYP27B1 in macrophages (Liu et al., 2006). This signal allows the macrophage to enhance vitamin D-mediated cathelicidin production even without high circulating calcitriol (Liu et al., 2006; Liu et al., 2007). However, previous work has shown that *Mycobacterium tuberculosis* (Mtb) lipoprotein LprE inhibits CYP27B1 and VDR upregulation, reducing cathelicidin production and enhancing bacterial survival (Padhi et al., 2019). This mechanism partially explains the persistence of tuberculosis within alveolar macrophages, and related bacteria might share a similar mechanism (Padhi et al., 2019).

*Mycobacterium avium* subsp. *paratuberculosis* (MAP) is known to cause Johne’s disease in ruminants, resulting in intestinal damage and chronic wasting (Rathnaiah et al., 2017). Furthermore, in some genetically susceptible patients, MAP infection causes Crohn’s disease (CD), an inflammatory bowel disease (IBD) characterized by asymmetrical, segmental, transmural inflammation with a relapsing-remitting pattern (Greenstein, 2003; Naser et al., 2004; Behr and Kapur, 2008; Rathnaiah et al., 2017). Similar to Mtb, MAP can infect macrophages and evade immune system clearance to establish a persistent infection, which warranted the necessity of using antibiotics for MAP eradication among infected CD patients (Perencevich and Burakoff, 2006; Arsenault et al., 2014; Qasem et al., 2016; Davis et al., 2017). Therefore, we were intrigued to find out if MAP shares Mtb’s method of evading immune detection by interfering with vitamin D signaling, which could be responsible for interference with *CAMP* expression and subsequent dysregulation of the intestinal microbiota in CD.

Additionally, therapeutic interventions of inactive vitamin D for IBD, which have so far shown mixed results for CD overall, might prove ineffective in MAP-infected patients and effective for MAP-uninfected patients. As such, it is necessary to determine whether MAP survival depends on interference with macrophage conversion of inactive calcifediol to active calcitriol, thereby inhibiting cathelicidin production and bacterial clearance. The objective of this study is to examine the effect of various forms of vitamin D and exogenous cathelicidin treatment on MAP infection and burden and subsequent macrophage-mediated inflammatory response. Our study clearly outlines a novel immunoevasive mechanism of MAP infection and reveals the importance of vitamin D signaling in eradicating infection in CD.

## Materials and Methods

### Measurement of Plasma Calcitriol and Cathelicidin in Clinical Samples

Plasma from peripheral blood samples (4.0 mL K_2_-EDTA tube) was collected from 100 CD patients (CDAI ≥220 and ≤450). The status of MAP infection was subsequently determined *via IS900* PCR as described earlier (Qasem et al., 2019), and then we randomly selected 40 MAP positive and 40 MAP negative CD patients for this study. We then used the Human Cathelicidin Antimicrobial Peptide ELISA Kit (MyBioSource, San Diego, CA) and the Calcitriol ELISA Kit (MyBioSource, San Diego, CA) to determine the plasma levels of cathelicidin and calcitriol, respectively. This study was approved by the University of Central Florida Institutional Review Board # STUDY00003468. All samples were de-identified before handling.

### Infection and Treatment of Monocyte-Derived Macrophages

The THP-1 cell line (ATCC TIB-202) was cultured in RPMI-1640 medium (ATCC 30-2001) with 10% fetal bovine serum (FBS; Sigma Life Science, St. Louis, MO). The cells were maintained in a humidified 5% CO_2_ incubator at 37°C and grown to confluency in cell culture flasks. A total of 1.0 mL of cell suspension was transferred to 12-well tissue culture plates with 1x10^5^ cells per well. They were then differentiated into monocyte-derived macrophages using 50 ng/mL phorbol 12-myristate 13-acetate (PMA; Sigma Life Science, St. Louis, MO) followed by 48 hours of incubation at 37°C. Next, monocyte-derived macrophages were treated with 5 µg/mL lipopolysaccharide (LPS) or infected with clinical MAP UCF4 (1x10^7^ CFU/mL), followed by 24 hours of incubation at the same conditions. When the macrophages were infected or stimulated with LPS, they also were dosed with 50 ng/mL vitamin D2, vitamin D3, calcifediol, or calcitriol, all purchased from Sigma Aldrich (St. Louis, MO), or 30 μg/mL LL-37 (Tocris Bioscience, Bristol, UK).

### Measurement of *CAMP, NOX-1, TNF-α, IL-1β*, and *IL-10* Expression in Treated Macrophages and Caco-2 Monolayers

RNA was isolated from each 1.0 mL sample of monocyte-derived macrophages following 24 hours of treatment with vitamin D or cathelicidin or from Caco-2 cells following 24 hours of co-culture with infected or treated macrophages. RNA was then reverse-transcribed to cDNA, then gene expression was measured using specific primers for *GAPDH, CAMP, TNF-α, IL-1β*, and *IL-10* obtained from Bio-rad (Hercules, CA) followed by quantitative reverse transcription PCR (RT-qPCR) analysis. RNA was extracted using the RNeasy ^®^ Mini Kit (Qiagen, Hilden, Germany) according to manufacturer protocols. RNA concentrations were measured using NanoDrop (OD at 260 nm). Next, cDNA was synthesized from 1000 ng of each RNA sample using 5.8 µL master mix made from the High Capacity cDNA Reverse Transcription Kit (Applied Biosystems, Waltham, MA) and then topped up to a total volume of 20 µL with RNase-free water, according to manufacturer protocols. A thermal cycler (MyGene Series Peltier Thermal Cycler) was used to perform the reactions for 5 min at 25°C, 20 min at 46°C, and 1 min at 95°C. The cDNA samples were stored at -20°C or used immediately for RT-qPCR analysis. For each sample, 5 µL of cDNA was mixed with 10 µL of Fast SYBR Green Master Mix (ThermoFisher Scientific, Waltham, MA), 1 µL primer mix, and 4 µL of DEPC-treated water. Samples were added in triplicate to a 96-well microamp RT-PCR reaction plate, and the experiment was run using 7500 Fast Real-Time PCR System (Applied Biosystems, Waltham, MA). Deleted: repeated sentence. GAPDH was the control used to obtain baseline CT readings. Relative mRNA expression levels were calculated using the equation (2^(- ΔΔCT).

### Measurement of LL-37, TNF-α, IL-1β, and IL-10 Protein Level in Treated Macrophages

Following 24 hours of infection and treatment with vitamin D forms or LL-37, monocyte-derived macrophages were pelleted by centrifugation at 2,500 rpm for 5 min at 4°C. The supernatants were saved, and TNF-α, IL-1β, and IL-10 protein levels were determined using the Ella automated immunoassay system (ProteinSimple, Santa Clara, CA). The Human Cathelicidin Antimicrobial Peptide ELISA Kit (MyBioSource, San Diego, CA) was used to determine LL-37 levels following manufacturer’s instructions.

### Measurement of MAP Viability in MGIT Culture

We inoculated 1 mL BACTEC™ MGIT™ ParaTB medium (BD Diagnostics, Sparks, MD) with 10^7 CFU/mL MAP strain UCF4 as described earlier (Qasem and Naser, 2018). The media was then treated with LL-37 (Tocris Bioscience, Bristol, UK) and Halt™ Protease Inhibitor Cocktail (Thermo Scientific, Rockford, IL). The same amount of protease inhibitor cocktail and LL-37 was added to the media every 3 days to maintain a consistent concentration. Bacterial growth expressed in CFU/mL was quantified daily using the BACTEC™ MGIT™ 320 for 20 consecutive days. The medium contains a molecule which fluoresces in the presence of actively respiring mycobacteria, permitting automatic quantification of growth as described previously (Qasem et al., 2016).

### Measurement of MAP Viability in Infected Macrophages

We cultured THP-1 macrophages in 2 mL media as described previously. Following 24 hours of MAP infection and vitamin D/LL-37 treatment, the cultures were treated with 350 µL lysis buffer (Qiagen, Hilden, Germany) and incubated at room temperature for 15 minutes. Subsequently, 700 μL of each sample were transferred to a respective 1.5 mL microcentrifuge tube, and all samples were centrifuged for 1 minute at 8,000 rcf. The pellet was resuspended by gently vortexing, and 100 μL of each sample was mixed with 100 μL BacTiter-Glo Microbial Cell Viability Assay (Promega, Madison, WI) in a 96 well opaque-sided plate. Samples were incubated at room temperature on a shaker for 5 minutes, and luminescence was recorded using the GloMax Navigator system GM-2000 (Promega, Madison, WI). Bacterial viability was analyzed from the generated luminescence.

### Measurement of Calcitriol Production in Treated Macrophages

Following 24 hours of infection with MAP and treatment with 50 ng/mL calcifediol, THP-1 monocyte-derived macrophages were pelleted by centrifugation at 2,500 rpm for 5 min at 4°C. The supernatants were saved, and calcitriol levels were determined using the Calcitriol ELISA Kit (MyBioSource, San Diego, CA).

### Knockdown of CAMP by siRNA Transfection

5 nmol of Silencer™ Pre-Designed siRNA (siRNA ID: 14402, ThermoFisher, Waltham, MA) specific to *CAMP* were diluted first in 50 uL nuclease-free H_2_O. 3.3 μL of this stock were mixed with 30 μL Optimem media (Gibco, Waltham, MA) and further diluted in an additional 450 μL Optimem. 27 μL Lipofectamine reagent (Invitrogen, Carlsbad, CA) was then mixed with 450 μL Optimem and the resulting mixture was added to the 459 μL of diluted siRNA mix. 300 μL of the resulting transfection master mix was added to every 2 mL of media containing target cells, or 15 μL into 100 μL of a 96 well plate.

### Co-Culturing THP-1 Macrophages With Caco-2 Monolayers

The effects of calcitriol and cathelicidin on macrophage-mediated oxidative stress were examined in a human enterocyte-like cell line (Caco-2 ATCC HTB-37). Cells were routinely cultured in ATCC-formulated Eagle’s Minimum Essential Medium (EMEM) supplemented with 20% FBS (ATCC, Manassas, VA) and maintained at 37°C in a humidified 5% CO_2_ incubator. Cells were grown in 12-well plates or microscope slides at a density of 3×10^5^ cells per well until confluency and differentiation were reached in 21 days. On day 14, THP-1 macrophages were plated separately in co-culture wells. They were differentiated, infected with MAP, and treated with vitamin D within these wells as previously described. 24 hours following the infection, the co-culture wells were transferred to the 12-well plates containing Caco-2 cells to permit the free exchange of cytokines and other paracrine signals.

### Visualizing Caco-2 Oxidative Stress *via* DHE Fluorescence Staining Assay

DHE fluorescence staining was performed on Caco-2 monolayers following 24 hours of co-culture with MAP-infected macrophages. First, monolayers were washed twice with cold PBS and then fixed with 4% paraformaldehyde (PFA) for 15 min. Monolayers were then washed twice with cold PBS and treated with 1 μM DHE stain (Sigma Aldrich, St. Louis, MO) for 25 min. Next, 60 μL VECTASHIELD Antifade Mounting Medium containing 4′,6-diamidino-2-phenylindole (DAPI; Vector Laboratories, Burlingame, CA) was used to co-stain nuclei. Slides were examined under Amscope IN480TC-FL-MF603 Fluorescence Microscope, where red staining indicates oxidative stress and blue staining represents nuclei. Captured images were analyzed by measuring average integrated density using NIH Image J 1.39o software, which was also used to generate merged images as we described earlier (Qasem et al., 2021).

### Measurement of Nicotinamide Adenine Dinucleotide Phosphate (NADPH)

Following 24 hours of co-culture with infected and treated macrophages, Caco-2 cells were lysed, and their levels of NADPH and total NADP were measured using the NADP/NADPH Assay Kit (Abcam, Cambridge, UK) according to manufacturer protocols. Briefly, the cell lysates were halved, with one half heated for 30 min to degrade the oxidized NADP+ while leaving the NADPH untouched. Each lysate was then mixed with the kit developer in triplicate on a 96 well plate and left to incubate in the dark for 24 hours. NADP was then quantified for each well, with the heated lysate measuring the reduced NADPH as a fraction of total NADP.

### Statistical Analysis

GraphPad Prism V.7.02 (GraphPad, La Jolla, CA, USA) was used for analyzing data statistics. The Kolmogorov–Smirnov normality test was used to test normal distribution for all values. Two-way analysis of variance (ANOVA) was used to assess significance among experiments, which was followed by Bonferroni correction test. Data are expressed as average ± SD of the mean, and the difference between treated samples vs. controls was considered statistically significant at a level of P-value < 0.05 and 95% confidence interval (CI). All experiments were performed in triplicates.

## Results

### Cathelicidin and Calcitriol Are Reduced in MAP-Infected CD Patients

**Figure 1:**
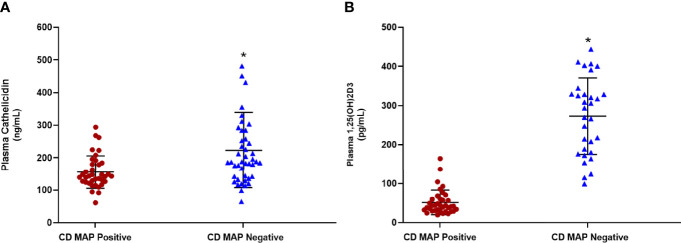
Levels of cathelicidin **(A)** and calcitriol (1,25(OH)2D3) **(B)** in clinical plasma samples obtained from MAP-positive CD and MAP-negative CD patients (N= 40 per group), were determined by the Human Cathelicidin Antimicrobial Peptide ELISA Kit (MyBioSource, San Diego, CA) and the Calcitriol ELISA Kit (MyBioSource, San Diego, CA). *Indicates P-value of less than 0.05.

We measured cathelicidin and calcitriol levels in 80 clinical plasma samples, 40 of which were MAP negative, 40 of which were MAP positive. We observed statistically significant reductions in plasma cathelicidin (**Figure 1A**) and plasma calcitriol (**Figure 1B**). Cathelicidin in MAP-positive patients measures 155.55 ± 49.77 ng/mL, increasing to 193.01 ± 78.95 ng/mL in MAP-negative patients. The shift in calcitriol levels was more dramatic than cathelicidin; the average calcitriol for MAP-positive patients was 51.48 ± 31.04 pg/mL, but 272.36 ± 94.77 pg/mL. This trend lends preliminary support to the hypothesis that MAP infection alters calcifediol hydroxylation.

### Calcitriol Enhances *CAMP* Expression and Cathelicidin Production in THP-1 Macrophages

**Figure 2:**
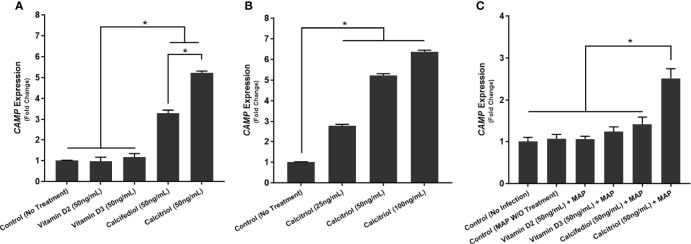
Effect of different forms of vitamin D **(A)** and different concentrations of calcitriol **(B)** on CAMP expression in the presence of MAP infection **(C)**. Relative expression was quantified using RT-qPCR. *Indicates P-value of less than 0.05.

**Figure 3:**
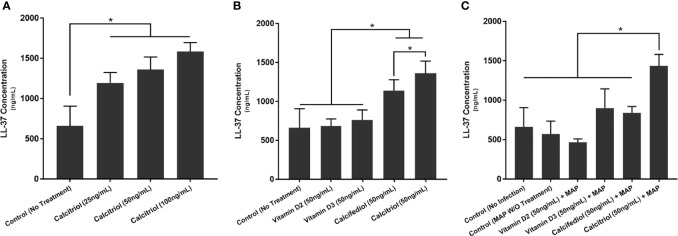
Effects of different forms of vitamin D **(A)** and different concentrations of calcitriol **(B)** on LL-37 production in the presence of MAP infection **(C)**. Cytokine concentration was quantified using the Ella system (ProteinSimple). *Indicates P-value of less than 0.05.

We next examined whether THP-1 macrophages respond to treatment with all forms of vitamin D or only calcitriol and calcifediol. Treatment with vitamin D2 and D3 did not significantly enhance *CAMP* expression compared with the control. Calcifediol treatment enhanced *CAMP* expression in uninfected cells by a factor of 3.29 ± 0.15, and calcitriol enhanced expression by a factor of 5.24 ± 0.08 (**Figure 2A**). The effect of calcitriol increased in a dose-dependent manner. Treatment with 25 ng/mL calcitriol enhanced expression by only 2.79 ± 0.07 fold, and 100 ng/mL yielded a 6.37 ± 0.07-fold change (**Figure 2B**). These phenomena change after MAP infection; MAP-infected cells had no significant *CAMP* enhancement upon treatment with calcifediol. However, calcitriol is still effective at increasing *CAMP* expression during MAP infection, increasing *CAMP* mRNA by a factor of 2.52 ± 0.23 (**Figure 2C**). These trends were later validated by ELISA, confirming that expression corresponds with LL-37 production (**Figure 3**).

### Calcitriol and LL-37 Significantly Reduce the Pro-Inflammatory Milieu Elicited by MAP-Infected Macrophages

**Figure 4:**
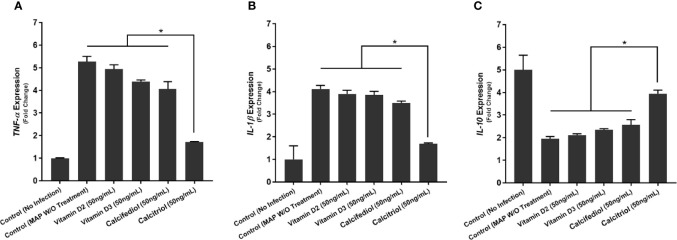
Effects of Vitamin D Treatment on the expression of TNF-α **(A)**, IL-1β **(B)**, and IL-10 **(C)** in MAP-infected macrophages. Relative expression was quantified using RT-qPCR. *Indicates P-value of less than 0.05.

To examine calcitriol’s effect on MAP-induced inflammation, we infected THP-1 macrophages with MAP and treated them with different forms of vitamin D. Calcitriol was the only form of vitamin D which significantly reduced TNF-α and IL-1β expression (**Figures 4A, B**) and production (**Table 1**) compared to the untreated, infected cells. Furthermore, calcitriol treatment partially rescued IL-10 expression (**Figure 4C**) and production (**Table 1**) in infected macrophages.

**Table 1:** Effect of Vitamin D treatment on cytokine production in MAP-infected macrophages.

Infection and Treatment	TNF-α ± SD	IL-1β ± SD	IL-10 ± SD
	(pg/mL)	(pg/mL)	(pg/mL)
Control (no infection)	60.52 ± 1.75	56.13 ± 1.81	125.65 ± 6.11
Control (MAP W/O Treatment)	173.12 ± 4.73	163.87 ± 5.72	69.18 ± 3.97
MAP + Vitamin D2	157.84 ± 3.14	145.37 ± 6.17	73.14 ± 3.69
MAP + Vitamin D3	151.11 ± 5.44	140.61 ± 5.74	76.95 ± 5.26
MAP + 25(OH)D	136.21 ± 2.48	127.54 ± 3.84	82.12 ± 2.55
MAP + 1,25(OH)2D3	81.66 ± 2.79*	74.39 ± 3.72*	105.28 ± 4.58*

*Indicates P-value of less than 0.05.

**Figure 5:**
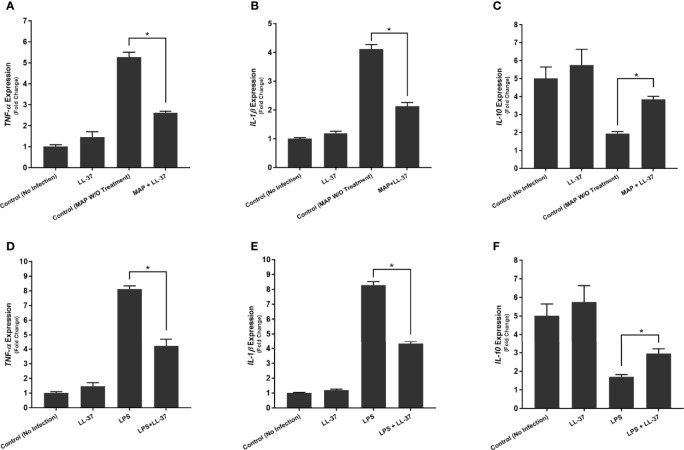
Effects of LL-37 treatment on the expression of TNF-α **(A, D)**, IL-1β **(B, E)**, and IL-10 **(C, F)** in MAP-infected **(A–C)** and LPS-stimulated **(D–F)** macrophages. Relative expression was quantified using RT-qPCR. *Indicates P-value of less than 0.05.

We observed similar effects with LL-37 treatment. MAP-infected macrophages showed a sharp decrease in TNF-α expression upon LL-37 treatment, a 5.27 ± 0.23-fold increase reduced to 2.61 ± 0.08 after treatment (**Figure 5A**). IL-1β showed a similar decrease with LL-37, a 4.12 ± 0.16-fold change reduced to 2.13 ± 0.13-fold (**Figure 5B**). IL-10, by contrast, increased in expression upon LL-37 treatment from 1.94 ± 0.11-fold to 3.85 ± 0.17-fold (**Figure 5C**). These trends were then verified by measuring cytokine production levels. TNF-α was secreted into the supernatant at concentrations of 173.12 ± 4.73 pg/mL upon MAP infection, but LL-37 treatment reduced it to 89.43 ± 4.96 pg/mL. Likewise, IL-1β secretion dropped from 163.87 ± 5.72 pg/mL to 82.23 ± 4.39 pg/mL and IL-10 secretion increased from 69.18 ± 3.69 pg/mL to 102.31 ± 4.11 pg/mL (**Table 2**). Interestingly, LPS-stimulated macrophages also decreased pro-inflammatory cytokine expression and increased IL-10 expression upon LL-37 treatment (**Figures 5D–F**). These results were verified by measuring cytokine production levels (**Table 2**). As such, LL-37 not only functions by clearing bacteria but can serve as an anti-inflammatory signal.

**Table 2:** Effects of LL-37 treatment on cytokine production in MAP-infected and LPS-stimulated macrophages.

Infection and Treatment	TNF-α ± SD	IL-1β ± SD	IL-10 ± SD
	(pg/mL)	(pg/mL)	(pg/mL)
Control (No Infection)	60.52 ± 1.75	56.13 ± 1.81	125.65 ± 6.11
LL-37	64.12 ± 2.83	61.42 ± 3.94	129.37 ± 3.82
Control (MAP W/O Treatment)	173.12 ± 4.73	163.87 ± 5.72	69.18 ± 3.69
MAP + LL-37	89.43 ± 4.96*	82.23 ± 4.39*	102.31 ± 4.11*
LPS Treatment	194.21 ± 3.51	183.04 ± 2.48	51.23 ± 1.56
LPS + LL-37	95.75 ± 2.64*	94.71 ± 1.75*	96.14 ± 6.41*

*Indicates P-value of less than 0.05.

### LL-37 Reduces MAP Viability in Both Bacterial Culture and Macrophages

**Figure 6:**
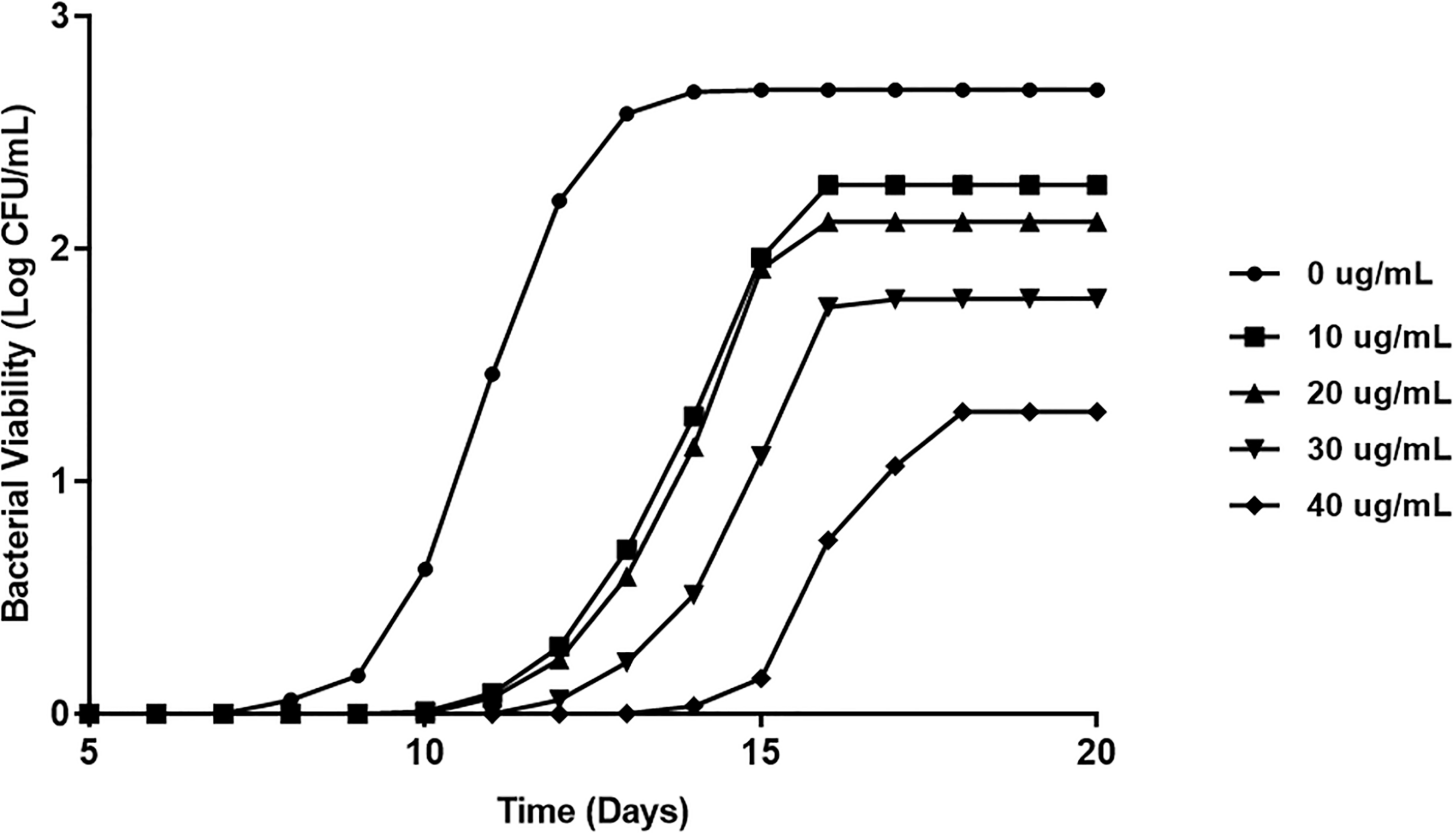
Direct effect of LL-37 treatment on MAP viability in MGIT culture. BD Bactec MGIT™ Para-TB medium (Becton Dickinson, Sparks, Maryland, USA) system was used to determine the *in vitro* inhibitory effect of LL-37 on MAP over 20 days. Bacterial viability was calculated using fluorescence quenching technology and by the BACTEC™ MGIT™ 320.

To verify that LL-37 reduces extracellular MAP viability, we inoculated five MGIT tubes with MAP and cultured them over the course of 20 days with differing concentrations of LL-37. We observed a concentration-dependent bacteriostatic effect of LL-37, by reducing both rate of growth and maximum bacterial load. At 50 ug/mL, MAP culture required an additional 3 days to reach the stationary phase, and bacterial load at stationary phase was far lower than the untreated culture (**Figure 6**).

**Figure 7:**
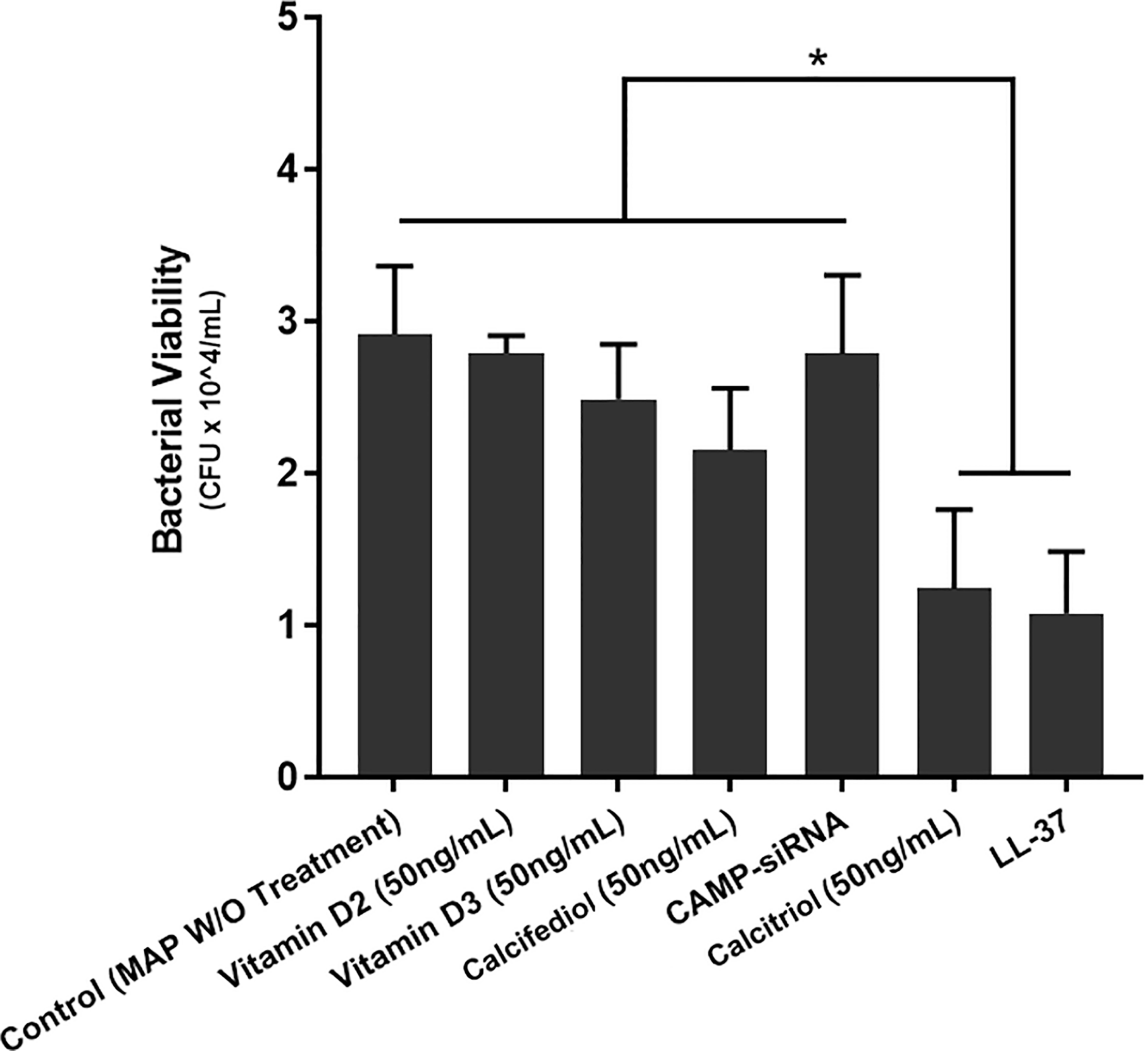
Effects of various vitamin D forms and LL-37 on intracellular MAP Viability in infected macrophages following 24 hours of treatment. Luminescence signal was detected using the GloMax Navigator system (Promega, GM-2000), and bacterial viability was analyzed from the generated luminescence. *Indicates P-value of less than 0.05.

Additionally, we tested the effects of multiple vitamin D forms and LL-37 on bacterial viability in MAP-infected macrophages. There was no significant change upon treatment with the inactive forms of vitamin D. However, both LL-37 treatment and calcitriol treatment substantially reduced MAP viability from 2.92 ± 0.45*10^4 CFU/mL to 1.07 ± 0.41*10^4 CFU/mL and 1.24 ± 0.52*10^4 CFU/mL, respectively (**Figure 7**).

### Knockdown of CAMP Eliminates the Anti-Inflammatory Effect of Calcitriol During MAP Infection

**Figure 8:**
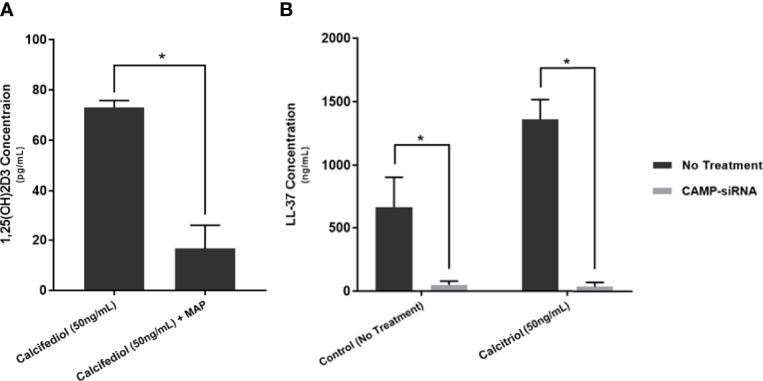
Calcitriol concentration following 24 of treating MAP-infected macrophages with 50 ng/mL of calcifediol **(A)**, and verification of successful cathelicidin knockdown by CAMP-siRNA **(B)**. Values were determined by Calcitriol ELISA Kit (MyBioSource, San Diego, CA) and the Human Cathelicidin Antimicrobial Peptide ELISA Kit (MyBioSource, San Diego, CA). *Indicates P-value of less than 0.05.

We treated two groups of macrophages with 50 ng/mL calcifediol and infected one group with MAP. Following 24 hours of infection, we collected the supernatant and measured calcitriol level. The uninfected macrophages yielded 72.98 ± 2.86 pg/mL calcitriol, while infected macrophages produced only 16.64 ± 9.23 pg/mL (**Figure 8A**). As such, the data indicate that MAP interferes with the conversion of calcifediol to calcitriol.

**Figure 9:**
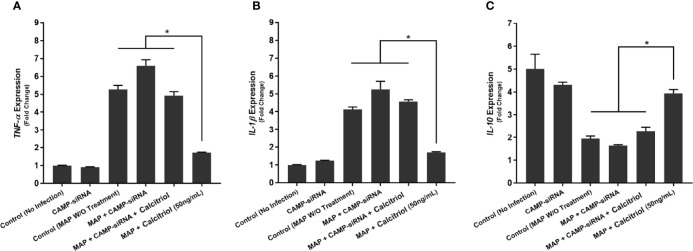
Effects of CAMP-siRNA transfection on the expression of TNF-α **(A)**, IL-1β **(B)**, and IL-10 **(C)** in MAP-infected macrophages. *Indicates P-value of less than 0.05.

Furthermore, we transfected THP-1 macrophages with CAMP-siRNA to inhibit cathelicidin translation while leaving other VDR-controlled genes unaffected (**Figure 8B**). We analyzed cytokine expression (**Figure 9**) and production (**Table 3**) in cells where *CAMP* was knocked down and calcitriol was present in the medium. *CAMP*-knockdown macrophages treated with calcitriol showed no significant reduction compared with untreated macrophages in TNF-α, IL-1β and IL-10 expression had no significant rescue. Accordingly, we conclude that during MAP infection, cathelicidin is necessary for calcitriol to mediate its anti-inflammatory effects.

**Table 3:** Effect of CAMP-siRNA transfection on cytokine production in MAP-infected macrophages.

Infection and Treatment	TNF-α ± SD	IL-1β ± SD	IL-10 ± SD
	(pg/mL)	(pg/mL)	(pg/mL)
Control (No Infection)	60.52 ± 1.75	56.13 ± 1.81	125.65 ± 6.11
CAMP-siRNA	69.07 ± 3.65	61.91 ± 2.63	120.46 ± 3.97
Control (MAP W/O Treatment)	173.12 ± 4.73	163.87 ± 5.72	69.18 ± 3.69
MAP + CAMP-siRNA	182.67 ± 3.18	173.75 ± 4.57	75.62 ± 5.26
MAP + CAMP-siRNA + 1,25(OH)2D3	144.18 ± 4.03	132.86 ± 5.76	80.19 ± 2.55
MAP + 1,25(OH)2D3	81.66 ± 2.79*	74.39 ± 3.72*	105.28 ± 4.58*

*Indicates P-value of less than 0.05.

### Calcitriol and Cathelicidin Reduce Macrophage-Mediated Oxidative Stress on Co-Cultured Caco-2 Monolayers

**Figure 10:**
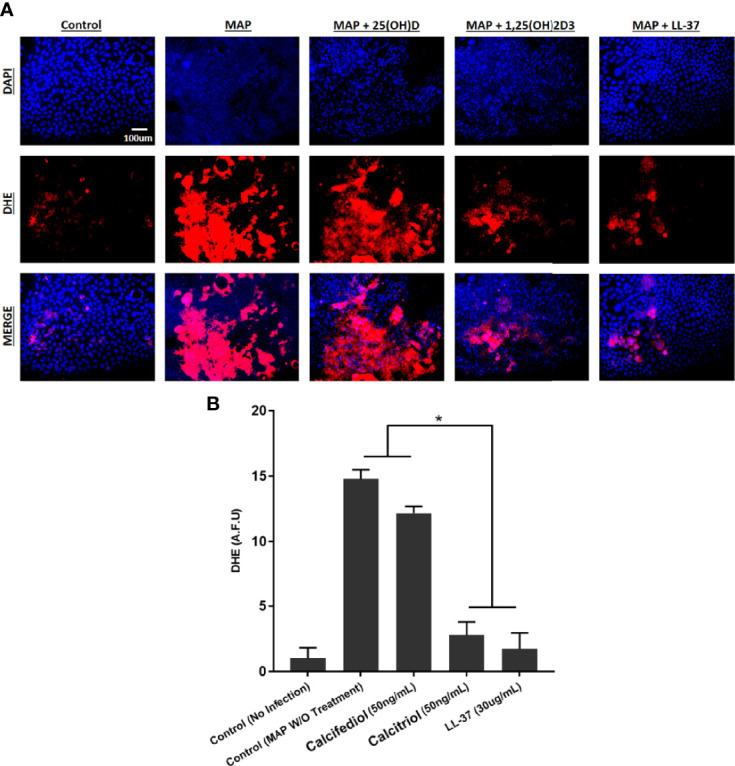
**(A)** The impact of calcitriol and LL-37 treatment on oxidative stress measured by co-cultured Caco-2 monolayers. Total nuclei are stained with DAPI in blue. DHE positive cells are stained in red, and merged cells are presented in pink. **(B)** Quantitative corrected DHE fluorescence integrated density from control and treated groups. *Indicates P-value of less than 0.05.

To examine the tissue damage effect of MAP-infected macrophages on co-cultured Caco-2 monolayers, we used three methods to assess oxidative stress levels. First, co-cultured Caco-2 monolayers were stained with DHE, imaged, and the red DHE stain was quantified using imageJ software (**Figure 10A**). Untreated MAP infection in co-cultured macrophages raised oxidative stress in the monolayer 14.78 ± 0.71 fold compared with the control. Treatment with LL-37 or calcitriol reduced oxidative stress to 1.74 ± 1.22 fold and 2.78 ± 1.00 fold, respectively (**Figure 10B**).

**Figure 11:**
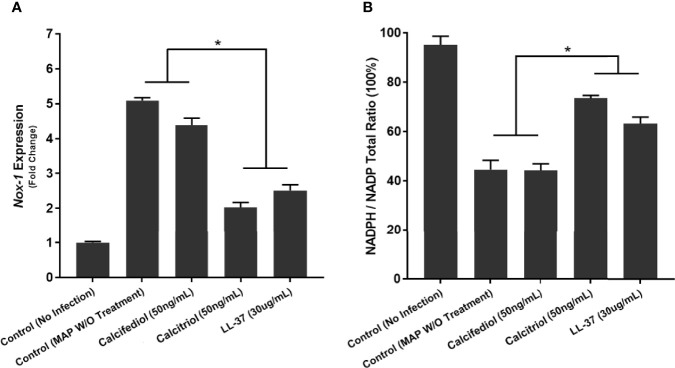
Quantitative Analysis of Oxidative Stress levels in Caco-2 Cells Co-Cultured with MAP-infected Macrophages following 24 hours of calcitriol or LL-37 treatment. Expression of *NOX-1* was quantified as fold change in comparison to Caco-2 cells co-cultured with uninfected macrophages **(A)**. Ratio of NADPH to NADP in lysate of Caco-2 cells expressed as a percentage **(B)** was measured using the NADP/NADPH Assay Kit (Abcam, Cambridge, UK). *Indicates P-value of less than 0.05.

We verified these results with analysis of *NOX-1* expression and NADPH/NADP assay in co-cultured Caco-2 monolayers. Expression of *NOX-1* was 5.09 ± 0.09 fold higher when the co-cultured macrophages went untreated, but calcitriol treatment reduced it to 2.03 ± 0.14 fold, and LL-37 reduced *NOX-1* expression to 2.51 ± 0.16 (**Figure 11A**). MAP infection in co-cultured macrophages caused a decline in NADPH/NADPt ratio to 44.51 ± 3.81%, indicating that a highly oxidative intracellular environment was present. Treating MAP-infected macrophages with LL-37 or calcitriol rescued NADPH to 63.12 ± 2.63% and 73.44 ± 1.17% of total NADP, respectively (**Figure 11B**).

## Discussion

Vitamin D deficiency is widespread in CD patients (Chatu et al., 2013; White, 2018). A recent meta-analysis found an inverse relationship between circulating vitamin D and CD severity (Sadeghian et al., 2016). Similarly, low vitamin D levels are inversely correlated with the likelihood of later surgical intervention in these patients (Ananthakrishnan et al., 2013). From a therapeutic standpoint, vitamin D supplementation has shown promise in reducing disease activity and inflammatory biomarkers (Guzman-Prado et al., 2020). However, little is known about how vitamin D is metabolized in patients with inflammatory bowel disease.

Vitamin D activation is necessary to mediate transcriptional changes (Christakos et al., 2016). It has been reported that vitamin D can directly inhibit the growth of bacteria following exposure to high doses, but the mechanism is unclear (Greenstein et al., 2012). Subversion of the antibacterial response is a classical and potent way for mycobacteria to evade the host immune response and establish persistent infection (Arsenault et al., 2014; Liu et al., 2017). Therefore, understanding how MAP alters the function of the macrophages in CD is crucial to explain why it is challenging to eradicate the infection in these patients. It is worth noting that Mtb possesses a host of mechanisms that assist in its survival within alveolar macrophages, many of which involve preventing phagolysosome fusion and halting apoptotic signals (Pecora et al., 2006; Jo, 2013). Interestingly, calcitriol has been shown to upregulate autophagy *via* cathelicidin, which leads to phagolysosome fusion and destruction of phagocytosed bacteria (Yuk et al., 2009; Shin et al., 2010). Similarly, a substantial body of work in cattle establishes MAP’s adept evasion of the bovine immune system (Arsenault et al., 2014).

Previous work has shown that Mtb possesses at least one protein that subverts the vitamin D signaling pathway in macrophages, altering the antibacterial response (Padhi et al., 2019). Here, we present evidence that MAP is similarly capable of affecting vitamin D activation. Our analysis of clinical samples has shown that calcitriol and cathelicidin are both reduced in MAP-positive CD patients compared with MAP-negative CD patients. Moreover, we demonstrated that both calcifediol and calcitriol induce expression and production of cathelicidin in uninfected macrophages, but MAP infection alters calcifediol’s inductive capacity. First, calcitriol treatment reduces pro-inflammatory cytokine expression and restores IL-10 production in MAP-infected macrophages. In addition, LL-37 treatment displayed similar effects to calcitriol, and we verified that LL-37 has potent anti-microbial effects against MAP in both bacterial culture and infected macrophages. Consequently, *CAMP* knockdown removes the beneficial effects of calcitriol and cathelicidin on MAP infection, which validated the role of LL-37 as a mediator of calcitriol’s anti-inflammatory signal in macrophages. Finally, we show that the anti-inflammatory effect of calcitriol and cathelicidin reduces MAP-induced oxidative stress in Caco-2 cells co-cultured with infected macrophages.

**Figure 12:**
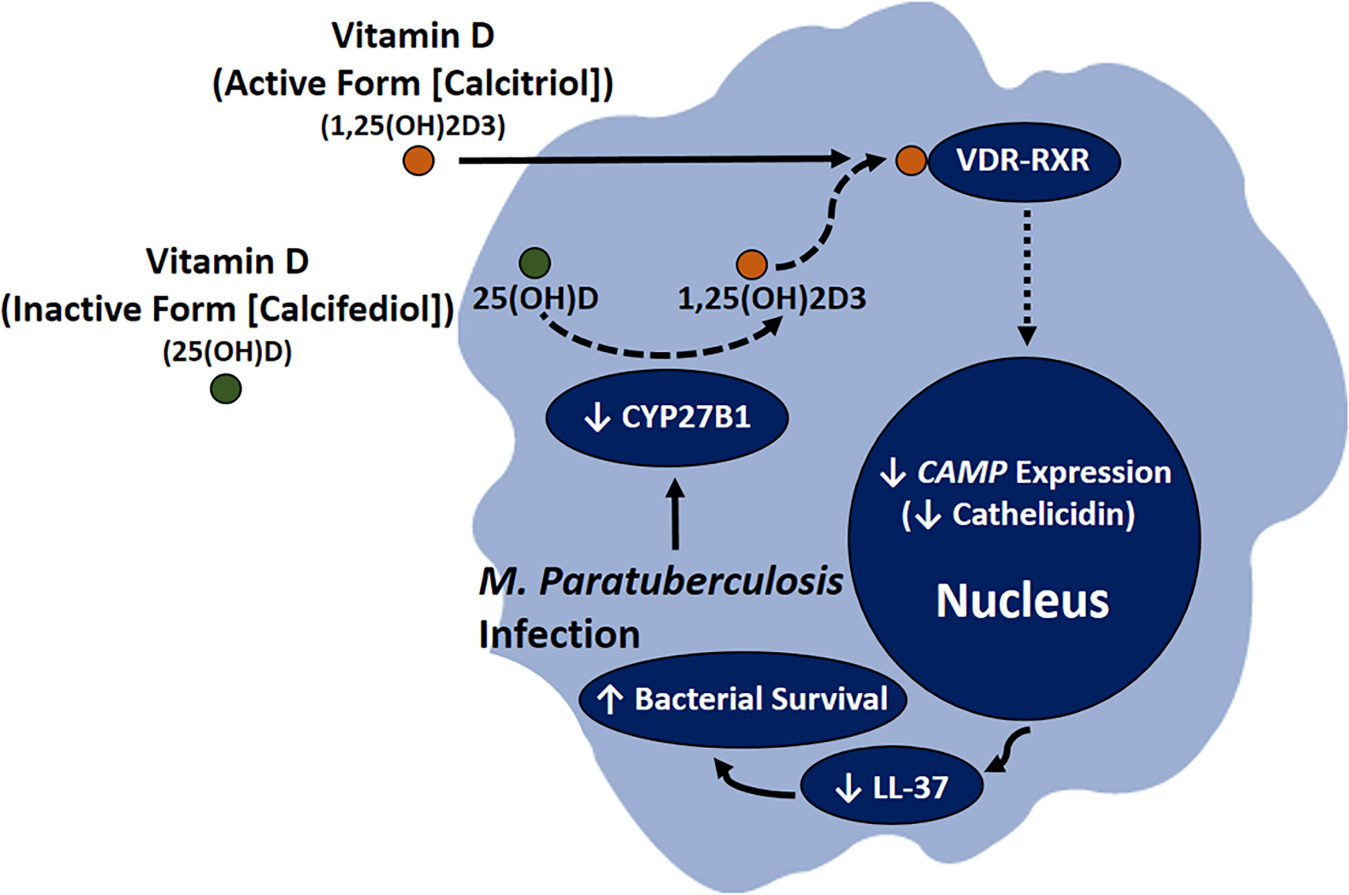
Role of MAP infection in CD pathogenesis through downregulating the conversion of calcifediol to calcitriol, resulting in lower *CAMP* expression, which leads to reduction in LL-37 production and increased bacterial viability in infected macrophages.

These findings strongly suggest that MAP and Mtb share a homologous mechanism that interferes with vitamin D signaling, which justifies further study on how MAP uniquely leads to CD pathogenesis. Additionally, our data highlight cathelicidin’s key role in mediating vitamin D’s anti-inflammatory properties and indicate that MAP substantially improves its viability by disrupting vitamin D signaling (**Figure 12**). Likewise, the inductive effect of cathelicidin on co-cultured epithelial cells suggests that this effect may correspond with reduced oxidative stress in intestinal tissue.

Further studies may determine if MAP suppresses calcitriol production in the same way as Mtb *via* a lipoprotein-mediated disruption of TLR2 signaling (Padhi et al., 2019). However, the lack of a comprehensive genomic map of any MAP strain may hamper the bioinformatics approach to examine homology between the two species. Nevertheless, impeded stimulation of this pathway would be compelling evidence of a homologous protein and could then direct protein isolation and purification studies.

Outside the context of immunity, vitamin D is a crucial signal for maintaining bone homeostasis (Christakos et al., 2016). Since IBD patients are at increased risk of osteoporosis and other skeletal abnormalities, an investigation into the mechanism by which IBD disrupts bone homeostasis is warranted (Andreassen et al., 1997). Previous work in our laboratory has identified distinct changes in undercarboxylated osteocalcin, activated osteocalcin, and serum calcium levels in MAP-infected bovines and CD patients (Naser et al., 2018). We have further noted a correlation between osteoporosis markers in the blood of rheumatoid arthritis (RA) patients, polymorphisms in the TNF-α gene and those of its receptor, and MAP infection (Naser et al., 2019). The findings of this study are highly suggestive of a novel mechanism by which MAP might interfere with bone homeostasis. An aberrant, prolonged inflammatory response paired with impaired vitamin D activation may account for MAP’s deleterious effect on CD and RA patients, where its presence would represent a subgroup at particular risk of osteoporosis. Consequently, testing for MAP DNA in RA and CD patients may prove valuable for clinicians.

From a therapeutic standpoint, MAP suppression of vitamin D activation suggests that the active form of vitamin D supplementation may prove more effective in MAP-infected CD patients since most of vitamin D commercial supplements are inactive (Christakos et al., 2016). This suggestion has some precedent in clinical trials with Mtb; despite vitamin D deficiency being a risk factor for tuberculosis, a course of supplementation with inactive vitamin D in Mongolian children had no significant impact on Mtb infection rates (Ganmaa et al., 2020). Furthermore, the fact that cathelicidin supplementation mirrors the effects of calcitriol on macrophage-mediated inflammation and enterocyte oxidative stress suggests that LL-37 could be a therapeutic option for the suppression of CD inflammatory symptoms. Accordingly, further studies of this phenomenon and vitamin D’s effect on CD patients are warranted.

## Data Availability Statement

The original contributions presented in the study are included in the article/supplementary material. Further inquiries can be directed to the corresponding author.

## Ethics Statement

The studies involving human participants were reviewed and approved by University of Central Florida Institutional Review Board # STUDY00003468. The patients/participants provided their written informed consent to participate in this study.

## Author Contributions

Conceptualization, AQ, JV, and SN. Formal analysis, JV and AQ. Funding acquisition, SN. Methodology, AQ, JV, and SN. Supervision, SN. Writing - original draft, JV. Writing - review and editing, AQ and SN. All authors have read and agreed to the submitted version of the manuscript.

## Funding

This study was supported in part by the Florida legislative grant.

## Conflict of Interest

The authors declare that the research was conducted in the absence of any commercial or financial relationships that could be construed as a potential conflict of interest.

## Publisher’s Note

All claims expressed in this article are solely those of the authors and do not necessarily represent those of their affiliated organizations, or those of the publisher, the editors and the reviewers. Any product that may be evaluated in this article, or claim that may be made by its manufacturer, is not guaranteed or endorsed by the publisher.

## Abbreviations

ANOVA, Analysis of variance; CAMP, Cathelicidin Antimicrobial Peptide; CD, Crohn’s disease; cDNA, complementary DNA; CI, Confidence interval; CYP27A1, Cytochrome P450 Family 27 Subfamily A Member 1; CYP27B1, Cytochrome P450 Family 27 Subfamily B Member 1; DHE, Dihydroethidium; DAPI, 4′,6-diamidino-2-phenylindole; ELISA, Enzyme-linked immunosorbent assay; EMEM, Eagle’s Minimum Essential Medium; GAPDH, Glyceraldehyde-3-phosphate dehydrogenase; IBD, Inflammatory Bowel Disease; IL-1β, Interleukin 1-beta; IL-10, Interleukin 10; LPS, Lipopolysaccharide; MAP, *Mycobacterium avium* subsp. *tuberculosis*; Mtb, *Mycobacterium tuberculosis;* NADP, Nicotinamide adenine dinucleotide phosphate; NOX-1, Nitric oxide synthase; PMA, Phorbol 12-myristate 13-acetate; PTH, Parathyroid hormone; RA, Rheumatoid arthritis; RT-qPCR, Reverse transcription quantitative real-time PCR; RXR, Retinoid X receptor; siRNA, Short interfering RNA; TLR, Toll-like receptor; TNF-α, Tumor necrosis factor alpha; VDR, Vitamin D receptor.

## References

[B1] Adams J. S. Rafison B. Witzel S. Reyes R. E. Shieh A. Chun R.. (2014). Regulation of the Extrarenal CYP27B1-Hydroxylase. J. Steroid Biochem. Mol. Biol. 144 Pt A, 22–27. doi: 10.1016/j.jsbmb.2013.12.009 24388948 PMC4077994

[B2] Alqasrawi D. Naser E. Naser S. A. (2021). Nicotine Increases Macrophage Survival Through α7nachr/NF-κb Pathway in Mycobacterium Avium Paratuberculosis Infection. Microorganisms 9. doi: 10.3390/microorganisms9051086 PMC8158352 34070119

[B3] Alqasrawi D. Qasem A. Naser S. A. (2020). Divergent Effect of Cigarette Smoke on Innate Immunity in Inflammatory Bowel Disease: A Nicotine-Infection Interaction. Int. J. Mol. Sci. 21. doi: 10.3390/ijms21165801 PMC7461043 32823518

[B4] Ananthakrishnan A. N. Cagan A. Gainer V. S. Cai T. Cheng S.-C. Savova G.. (2013). Normalization of Plasma 25-Hydroxy Vitamin D Is Associated With Reduced Risk of Surgery in Crohn’s Disease. Inflamm. Bowel Dis. 19, 1921–1927. doi: 10.1097/MIB.0b013e3182902ad9 23751398 PMC3720838

[B5] Andreassen H. Rungby J. Dahlerup J. F. Mosekilde L. (1997). Inflammatory Bowel Disease and Osteoporosis. Scand. J. Gastroenterol. 32, 1247–1255. doi: 10.3109/00365529709028155 9438324

[B6] Arsenault R. J. Maattanen P. Daigle J. Potter A. Griebel P. Napper S. (2014). From Mouth to Macrophage: Mechanisms of Innate Immune Subversion by Mycobacterium Avium Subsp. Paratuberculosis. Vet. Res. 45, 54. doi: 10.1186/1297-9716-45-54 24885748 PMC4046017

[B7] Behr M. A. Kapur V. (2008). The Evidence for Mycobacterium Paratuberculosis in Crohn’s Disease. Curr. Opin. Gastroenterol. 24, 17–21. doi: 10.1097/MOG.0b013e3282f1dcc4 18043227

[B8] Carlberg C. (2019). Vitamin D Signaling in the Context of Innate Immunity: Focus on Human Monocytes. Front. Immunol. 10, 2211. doi: 10.3389/fimmu.2019.02211 31572402 PMC6753645

[B9] Chang S. W. Lee H. C. (2019). Vitamin D and Health - The Missing Vitamin in Humans. Pediatr. Neonatol. 60, 237–244. doi: 10.1016/j.pedneo.2019.04.007 31101452

[B10] Chatu S. Chhaya V. Holmes R. Neild P. Kang J. Y. Pollok R. C.. (2013). Factors Associated With Vitamin D Deficiency in a Multicultural Inflammatory Bowel Disease Cohort. Frontline Gastroenterol. 4, 51–56. doi: 10.1136/flgastro-2012-100231 28839700 PMC5369785

[B11] Christakos S. Dhawan P. Verstuyf A. Verlinden L. Carmeliet G. (2016). Vitamin D: Metabolism, Molecular Mechanism of Action, and Pleiotropic Effects. Physiol. Rev. 96, 365–408. doi: 10.1152/physrev.00014.2015 26681795 PMC4839493

[B12] Davis W. C. Kuenstner J. T. Singh S. V. (2017). Resolution of Crohn’s (Johne’s) Disease With Antibiotics: What Are the Next Steps? Expert Rev. Gastroenterol. Hepatol. 11, 393–396. doi: 10.1080/17474124.2017.1300529 28276276

[B13] El-Sharkawy A. Malki A. (2020). Vitamin D Signaling in Inflammation and Cancer: Molecular Mechanisms and Therapeutic Implications. Molecules 25. doi: 10.3390/molecules25143219 PMC7397283 32679655

[B14] Ganmaa D. Uyanga B. Zhou X. Gantsetseg G. Delgerekh B. Enkhmaa D.. (2020). Vitamin D Supplements for Prevention of Tuberculosis Infection and Disease. New Engl. J. Med. 383, 359–368. doi: 10.1056/NEJMoa1915176 32706534 PMC7476371

[B15] Greenstein R. J. (2003). Is Crohn’s Disease Caused by a Mycobacterium? Comparisons With Leprosy, Tuberculosis, and Johne’s Disease. Lancet Infect. Dis. 3, 507–514. doi: 10.1016/S1473-3099(03)00724-2 12901893

[B16] Greenstein R. J. Su L. Brown S. T. (2012). Vitamins A & D Inhibit the Growth of Mycobacteria in Radiometric Culture. PloS One 7, e29631. doi: 10.1371/journal.pone.0029631 22235314 PMC3250462

[B17] Guzman-Prado Y. Samson O. Segal J. P. Limdi J. K. Hayee B. (2020). Vitamin D Therapy in Adults With Inflammatory Bowel Disease: A Systematic Review and Meta-Analysis. Inflamm. Bowel Dis. 26, 1819–1830. doi: 10.1093/ibd/izaa087 32385487

[B18] Jo E.-K. (2013). Autophagy as an Innate Defense Against Mycobacteria. Pathog. Dis. 67, 108–118. doi: 10.1111/2049-632X.12023 23620156

[B19] Liu C. H. Liu H. Ge B. (2017). Innate Immunity in Tuberculosis: Host Defense vs Pathogen Evasion. Cell Mol. Immunol. 14, 963–975. doi: 10.1038/cmi.2017.88 28890547 PMC5719146

[B20] Liu P. T. Stenger S. Li H. Wenzel L. Tan B. H. Krutzik S. R.. (2006). Toll-Like Receptor Triggering of a Vitamin D-Mediated Human Antimicrobial Response. Science 311, 1770–1773. doi: 10.1126/science.1123933 16497887

[B21] Liu P. T. Stenger S. Tang D. H. Modlin R. L. (2007). Cutting Edge: Vitamin D-Mediated Human Antimicrobial Activity Against Mycobacterium Tuberculosis Is Dependent on the Induction of Cathelicidin. J. Immunol. 179, 2060–2063. doi: 10.4049/jimmunol.179.4.2060 17675463

[B22] Naser S. A. Ghobrial G. Romero C. Valentine J. F. (2004). Culture of Mycobacterium Avium Subspecies Paratuberculosis From the Blood of Patients With Crohn’s Disease. Lancet 364, 1039–1044. doi: 10.1016/S0140-6736(04)17058-X 15380962

[B23] Naser A. Odeh A. K. Sharp R. C. Qasem A. Beg S. Naser S. A. (2019). Polymorphisms in TNF Receptor Superfamily 1b (TNFRSF1B:rs3397) Are Linked to Mycobacterium Avium Paratuberculosis Infection and Osteoporosis in Rheumatoid Arthritis. Microorganisms 7. doi: 10.3390/microorganisms7120646 PMC6955732 31817071

[B24] Naser A. Qasem A. Naser S. A. (2018). Mycobacterial Infection Influences Bone Biomarker Levels in Patients With Crohn’s Disease. Can. J. Physiol. Pharmacol. 96, 662–667. doi: 10.1139/cjpp-2017-0700 29638140

[B25] Padhi A. Pattnaik K. Biswas M. Jagadeb M. Behera A. Sonawane A. (2019). Mycobacterium Tuberculosis LprE Suppresses TLR2-Dependent Cathelicidin and Autophagy Expression to Enhance Bacterial Survival in Macrophages. J. Immunol. 203, 2665–2678. doi: 10.4049/jimmunol.1801301 31619537

[B26] Pecora N. D. Gehring A. J. Canaday D. H. Boom W. H. Harding C. V. (2006). Mycobacterium Tuberculosis LprA Is a Lipoprotein Agonist of TLR2 That Regulates Innate Immunity and APC Function. J. Immunol. 177, 422–429. doi: 10.4049/jimmunol.177.1.422 16785538

[B27] Perencevich M. Burakoff R. (2006). Use of Antibiotics in the Treatment of Inflammatory Bowel Disease. Inflamm. Bowel Dis. 12, 651–664. doi: 10.1097/01.MIB.0000225330.38119.c7 16804403

[B28] Qasem A. Naser S. A. (2018). Tnfα Inhibitors Exacerbate Mycobacterium Paratuberculosis Infection in Tissue Culture: A Rationale for Poor Response of Patients With Crohn’s Disease to Current Approved Therapy. BMJ Open Gastroenterol. 5, e000216. doi: 10.1136/bmjgast-2018-000216 PMC6067372 30073091

[B29] Qasem A. Naser A. E. Naser S. A. (2021). Enteropathogenic Infections Modulate Intestinal Serotonin Transporter (SERT) Function by Activating Toll-Like Receptor 2 (TLR-2) in Crohn’s Disease. Sci. Rep. 11, 22624. doi: 10.1038/s41598-021-02050-3 34799637 PMC8604993

[B30] Qasem A. Ramesh S. Naser S. A. (2019). Genetic Polymorphisms in Tumour Necrosis Factor Receptors (TNFRSF1A/1B) Illustrate Differential Treatment Response to Tnfα Inhibitors in Patients With Crohn’s Disease. BMJ Open Gastroenterol. 6, e000246. doi: 10.1136/bmjgast-2018-000246 PMC6361334 30815272

[B31] Qasem A. Safavikhasraghi M. Naser S. A. (2016). A Single Capsule Formulation of RHB-104 Demonstrates Higher Anti-Microbial Growth Potency for Effective Treatment of Crohn’s Disease Associated With Mycobacterium Avium Subspecies Paratuberculosis. Gut Pathog. 8, 45. doi: 10.1186/s13099-016-0127-z 27708718 PMC5041445

[B32] Rathnaiah G. Zinniel D. K. Bannantine J. P. Stabel J. R. Gröhn Y. T. Collins M. T.. (2017). Pathogenesis, Molecular Genetics, and Genomics of Mycobacterium Avium Subsp. Paratuberculosis, the Etiologic Agent of Johne’s Disease. Front. Vet. Sci. 4, 187. doi: 10.3389/fvets.2017.00187 29164142 PMC5681481

[B33] Sadeghian M. Saneei P. Siassi F. Esmaillzadeh A. (2016). Vitamin D Status in Relation to Crohn’s Disease: Meta-Analysis of Observational Studies. Nutrition 32, 505–514. doi: 10.1016/j.nut.2015.11.008 26837598

[B34] Shin D.-M. Yuk J.-M. Lee H.-M. Lee S.-H. Son J. W. Harding C. V.. (2010). Mycobacterial Lipoprotein Activates Autophagy *via* TLR2/1/CD14 and a Functional Vitamin D Receptor Signalling. Cell. Microbiol. 12, 1648–1665. doi: 10.1111/j.1462-5822.2010.01497.x 20560977 PMC2970753

[B35] Tabatabaeizadeh S.-A. Tafazoli N. Ferns G. A. Avan A. Ghayour-Mobarhan M. (2018). Vitamin D, the Gut Microbiome and Inflammatory Bowel Disease. J. Res. Med. Sci.: Off. J. Isfahan Univ. Med. Sci. 23, 75–75. doi: 10.4103/jrms.JRMS_606_17 PMC6116667 30181757

[B36] Vandamme D. Landuyt B. Luyten W. Schoofs L. (2012). A Comprehensive Summary of LL-37, the Factotum Human Cathelicidin Peptide. Cell. Immunol. 280, 22–35. doi: 10.1016/j.cellimm.2012.11.009 23246832

[B37] White J. H. (2018). Vitamin D Deficiency and the Pathogenesis of Crohn’s Disease. J. Steroid Biochem. Mol. Biol. 175, 23–28. doi: 10.1016/j.jsbmb.2016.12.015 28025175

[B38] Yuk J.-M. Shin D.-M. Lee H.-M. Yang C.-S. Jin H. S. Kim K.-K.. (2009). Vitamin D3 Induces Autophagy in Human Monocytes/Macrophages *via* Cathelicidin. Cell Host Microbe 6, 231–243. doi: 10.1016/j.chom.2009.08.004 19748465

